# Novel *FBN1* intron variant causes isolated ectopia lentis via in-frame exon skipping

**DOI:** 10.1038/s10038-025-01318-0

**Published:** 2025-02-13

**Authors:** Norihiro Shimizu, Yoichi Mashimo, Hirotaka Yokouchi, Yosuke Nishio, Setsu Sawai, Tomohiko Ichikawa, Tomoo Ogi, Takayuki Baba, Yoshihiro Onouchi

**Affiliations:** 1 Department of Ophthalmology and Visual Science, Chiba University Graduate School of Medicine, Chiba, Japan; 2 Maebara Shimizu Eye Clinic, Funabashi, Japan; 3 Department of Public Health, Chiba University Graduate School of Medicine, Chiba, Japan; 4 Department of Ophthalmology, Teikyo University Chiba Medical Center, Ichihara, Japan; 5 Department of Pediatrics, Nagoya University Graduate School of Medicine, Nagoya, Japan; 6 Department of Genetics, Research Institute of Environmental Medicine, Nagoya University, Nagoya, Japan; 7 Department of Neurology, Chiba Aoba Municipal Hospital, Chiba, Japan; 8 Division of Clinical Genetics, Chiba University Hospital, Chiba, Japan; 9 Department of Urology, Chiba University Graduate School of Medicine, Chiba, Japan; 10 Department of Human Genetics and Molecular Biology, Graduate School of Medicine, Nagoya University, Nagoya, Japan; 11 Center for One Medicine Innovative Translational Research (COMIT), Nagoya University Institute for Advanced Study, Nagoya, Japan; 12 Division of Molecular Physiology and Dynamics, Institute for Glyco-core Research (iGCORE), Tokai National Higher Education and Research System, Nagoya, Japan

**Keywords:** Disease genetics, Diseases

## Abstract

Mutations in *fibrillin-1* (*FBN1*) cause various clinical conditions, such as Marfan syndrome (MFS). However, the genotype–phenotype relationships underlying MFS and other conditions relevant to *FBN1* mutations have not been fully elucidated. We performed whole-exome sequencing on three participants, including an affected mother–daughter pair, in a three-generation Japanese family with isolated ectopia lentis (IEL). The sequencing identified a novel single-nucleotide variant (c.1327+3A>C) in intron 11 of *FBN1* that was shared between the two patients. We confirmed the co-segregation of the variant with IEL in two additional affected relatives in the family. The Combined Annotation-Dependent Depletion score of the variant was 26.1, which was indicated by SpliceAI to influence splicing, with a score of 0.93. Reverse transcription-polymerase chain reaction (RT-PCR) of mRNAs isolated from peripheral blood mononuclear cells revealed aberrant bands in all four affected individuals. Subsequent sequencing revealed that these bands originated from *FBN1* transcripts lacking exon 11. The causality of the variant in the skipping of exon 11, which results in an in-frame deletion of 60 amino acids corresponding to the “hinge” region of FBN1 protein, was confirmed in a minigene experiment. Interestingly, the same result was observed for a minigene for c.1327+1G>A, a variant previously identified in two unrelated EL families without MFS manifestations. These results suggest that the c.1327+3A>C mutation in *FBN1* likely leads to IEL. The findings expand our knowledge of FBN1 and provide insights into FBN1-related diseases.

## Introduction

Ectopia lentis (EL) is the dislocation or displacement of the eye lens. EL can cause significant intraocular complications, such as pupillary block glaucoma, retinal damage, and blindness. Surgical treatments for EL are becoming well-established. EL can occur after trauma, as a systemic manifestation of pseudoexfoliation syndrome, or as a result of a genetic disorder. *FBN1* is one of the genes causing familial EL and, patients with no phenotype in organs other than the eyes are termed isolated EL (IEL; Online Mendelian Inheritance in Man [OMIM] entry #129600) [1, 2]. *FBN1* encodes fibrillin-1, a structural protein that polymerizes into microfibrils. Fibrillin microfibrils are morphologically characteristic fibrils present in all connective tissues and assemble into tissue-specific structural frameworks. Marfan syndrome (MFS; OMIM #154700) is a typical inherited connective tissue disease caused by mutations in *FBN1* that shows autosomal dominant (AD) inheritance. MFS is characterized by skeletal abnormalities (tall stature and long arms, legs, fingers, and toes), lens abnormalities represented by EL, and thoracic aortic aneurysm and dissection. The prevalence of MFS is approximately 1.5–20 in 100,000 [3], with *FBN1* mutations detected in 66–91% of patients with MFS [4, 5]. EL is a major symptom of MFS; approximately 60% of all patients with MFS exhibit EL [6]. Ghent nosology, the current diagnostic criteria for MFS, places significant emphasis on aortic root aneurysms and EL [7]. Weill–Marchesani syndrome 2 (WMS2; OMIM #698328), characterized by short stature and brachydactyly, is another rare AD disorder (prevalence of 1 in 100,000) caused by mutations in *FBN1*. El is one of the main symptoms of WMS2 [8]. Additionally, mutations in *FBN1* have been implicated in other disorders, including Marfan lipodystrophy syndrome (OMIM #616914) [9], MASS phenotype (mitral valve prolapse, aortic dilatation without dissection, skeletal and skin abnormalities) (OMIM #604308) [10], stiff skin syndrome (OMIM #184900) [11], and acromicric dysplasia (OMIM #102370) [12]. Over 1000 disease-causing *FBN1* mutations have been reported. However, the correlation between genotypes and phenotypes remains unelucidated [13–16].

In 1995, the human *FBN1* mutation database, UMD-FBN1 (www.umd.be/), was created to standardize information on *FBN1* mutations, facilitating mutation analysis and identification of structure–function, and phenotype–genotype relationships. According to this database, 1847 different mutations have been identified in the *FBN1* sequence, including 51 (1.65%) large rearrangements. Of these, 200 splice mutations have been reported [17].

In this study, we describe a novel mutation within an intron of *FBN1* that is expected to co-segregate with IEL and cause splicing abnormalities based on evidence from three generations of an affected family.

## Materials and methods

### Study participants

**Fig. 1:**
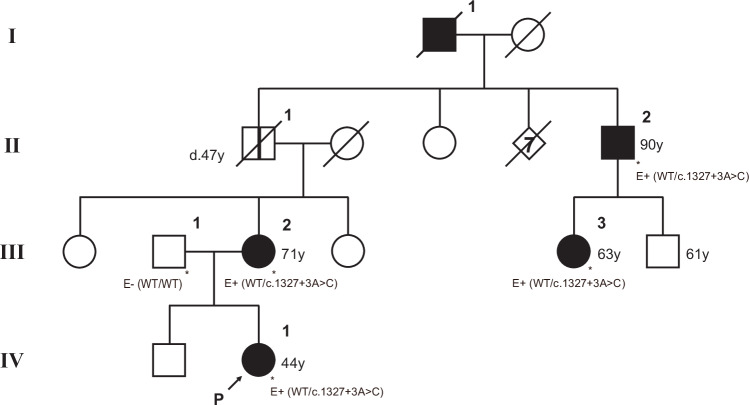
Pedigree of the studied family. The distribution of the affected individuals in the family suggests an autosomal dominant mode of inheritance. It is unknown whether patient I-1 had been diagnosed with lens dislocation, but it seems reasonable to assume that he was the founder of the causative mutation in this family and that the acute ocular hypertension that caused blindness was due to EL, as were several descendants. Patient II-1, who died at 47 years of age due to systemic infection, had no ophthalmological abnormalities

The proband (IV-1) was a 44-year-old Japanese woman diagnosed with EL at our institution. In the same year, her mother’s paternal cousin (III-3) developed EL at the age of 62. An interview regarding their family history strongly suggested that the proband’s mother (III-2) and maternal great uncle (II-2) had a history reminiscent of EL. In addition, the proband’s maternal great-grandfather (I-1), who had already died, was blind due to acute ocular hypertension (Fig. 1). Assuming familial EL with AD inheritance, four members of this single pedigree affected or suspected to be affected by EL (II-2, III-2, III-3, and IV-1), and one unaffected male (III-1) were recruited for the genetic study.

### Medical evaluation of the participants

An ophthalmologist, cardiologist, and orthopedist clinically evaluated the participants for the presence of symptoms, particularly those observed in the MFS and WMS (Supplementary Table 1).

### Identification of genetic variants

Genomic DNA was extracted from the peripheral blood leukocytes of the five participants using the Gentra Puregene Blood Kit (QIAGEN, Venlo, Netherlands) according to the manufacturer’s instructions. To identify the underlying genetic cause of the disease, we first performed whole-exome sequencing (WES) on the proband (IV-1), affected mother (III-2), and unaffected father (III-1). WES was performed at the Nagoya University Initiative on Rare and Undiagnosed Diseases Analysis Center using SureSelect Human All Exon V6 and Illumina HiSeq X, following the procedure described in our previous paper [18]. Subsequently, candidate variants identified by WES were examined in other affected members (II-2 and III-3) using polymerase chain reaction (PCR) direct sequencing. The Pr1 PCR primer set (Supplementary Table 2) was also used for direct sequencing. The sequencing reaction was performed using a BigDye Terminator v3.1 Cycle Sequencing Kit (Thermo Fisher Scientific, Waltham, MA), and the products were separated using an ABI 3130xl Genetic Analyzer (Thermo Fisher Scientific). The deleteriousness of the identified variants was assessed using the Combined Annotation-Dependent Depletion (CADD) tool GRCh38-v1.6 [19, 20]. To identify the intronic variants, we referred to the “SpliceAI” (https://github.com/Illumina/SpliceAI) and “MMSplice” (https://github.com/gagneurlab/MMSplice_MTSplice) scores within the CADD tool.

### Identification of aberrant splicing

Total RNA was extracted from peripheral blood mononuclear cells (PBMCs) of the four affected individuals using NucleoSpin RNA (TaKaRa Bio, Inc., Kusatsu, Japan) and reverse transcribed using ReverTra Ace qPCT RT Master Mix (TOYOBO, Osaka, Japan). After amplifying *FBN1* cDNA using forward and reverse primers created on exons 10 and 12, respectively (Pr2 in Supplementary Table 2), the amplicons were separated on an agarose gel. The separated amplicons were stained with Midori Green Advance DNA stain solution (Nippon Genetics, Tokyo, Japan), excised using a blue-green LED transilluminator, extracted from the gels, and sequenced using the Sanger method.

### *FBN1* minigene model

To investigate the effect of c.1327+3A>C on pre-mRNA splicing, we created plasmid constructs expressing minigenes of *FBN1*. First, a wild-type (WT) amplicon of a 5,348-base-pair (bp) genomic DNA fragment from exons 10 to 12 of the *FBN1* gene was cloned into the mammalian expression vector pcDNA3.1(+) using an In-Fusion HD Cloning Kit (TaKaRa Bio, Inc.). Subsequently, a mutant (MT) minigene construct for c.1327+3A>C was created by PCR-based mutagenesis using PrimeSTAR Max DNA Polymerase (TaKaRa Bio, Inc.). For comparison, we created a construct for c.1327+1G>A, a variant previously identified in patients with EL [21, 22] The primer sequences used for cloning and mutagenesis are listed in Supplementary Table 2. Modification of the intended nucleotides in these constructs was confirmed using Sanger sequencing.

HEK293 cells were maintained in a minimum essential medium supplemented with 10% fetal bovine serum, 1% non-essential amino acids, and 1% antibiotic–antimycotics (Thermo Fisher Scientific). Cells were seeded in six-well plates one day before transfection and transiently transfected with 0.5 μg of the WT or MT *FBN1* minigene construct using Lipofectamine LTX combined with Plus Reagent (Thermo Fisher Scientific) after replacement of the serum-free medium. Cells were harvested after 48 h of cultivation, and RNA was extracted. Splicing of pre-mRNAs expressed from the minigenes was analyzed using reverse transcription-PCR (RT-PCR) and agarose gel electrophoresis.

## Results

### Clinical features

The clinical characteristics of four family members with a history of EL were examined. First, the participants underwent detailed ophthalmological examinations and interviews. Patients II-2 and III-2 who had undergone intraocular lens (IOL) implantation at a medical institute in Chiba Prefecture were referred to our hospital for systemic evaluation and genetic counseling. II-2 was diagnosed with ocular hypertension and lens dislocation in the right eye at 57-years-of-age and underwent surgery for lens removal and IOL fixation. Seven years later, he developed visual difficulties in the left eye. He was determined to have decreased corneal endothelial cells in his left eye and received lens removal and IOL fixation. Because the patient was elderly and each surgery had been performed at different facilities, not at the referring hospital, and more than 30 years ago, no detailed information on the patient’s condition between the onsets and surgeries was available. However, given the patient’s history of laser iridotomy in both eyes, we speculated that he experienced acute ocular hypertension due to EL, which was initially treated as narrow-angle and ocular hypertension of unknown cause or an acute angle-closure glaucoma attack. The patient’s left eye exhibited bullous keratopathy. Currently, the corrected visual acuity is 0.7/0.1, and intraocular pressure (IOP) is 13/14 mmHg.

Patient III-2 developed lens dislocations in the right and left eyes at 55 and 56 years of age, respectively, and underwent IOL fixation at a referring hospital. Currently, the corrected visual acuity is 1.2/1.2, and the IOP is 20/19 mmHg.

**Fig. 2:**
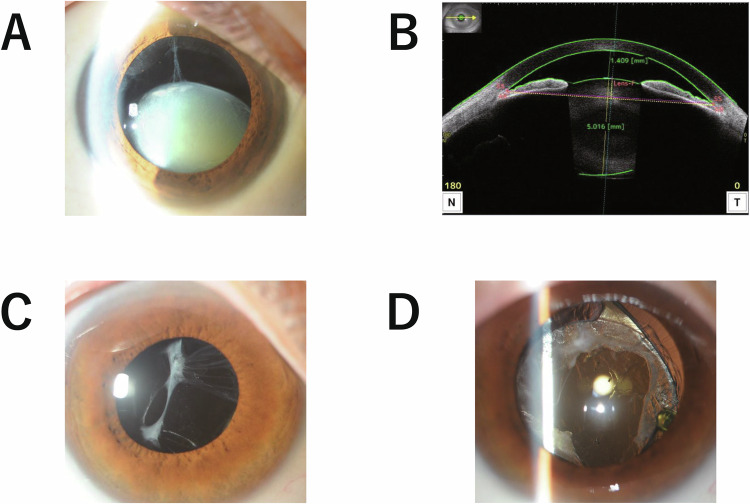
Images of the eyes of the affected individuals. **A** Right anterior eye of III-3 with ectopia lentis and the lens dislocated downwards. **B** Optical coherence tomography of the anterior segment of the left eye of patient IV-1 upon referral to our hospital. The anterior lens deviated, and the anterior chamber depth was as short as 1.46 mm. **C** Sac shrinkage in the right eye of patient IV-1 4 months after cataract surgery. The patient subsequently underwent YAG laser capsulotomy. **D** Right eye of patient IV-1. Partial tearing of the ciliary zonule and deviation of the lens capsule was clearly observed soon after YAG laser capsulotomy

Patient III-3 experienced sudden vision impairment in the left eye, was diagnosed with an acute glaucoma attack at a local ophthalmologic clinic and was referred to our hospital at 55 years of age. Soon after admission, the lens in the left eye dislocated into the vitreous cavity. The patient underwent IOL suturing at our hospital. At 62 years of age, she developed inferior lens dislocation in the right eye (Fig. 2A) and underwent intrascleral IOL fixation at our hospital. Her dislocated lens had Emery-Little grade 2 cloudiness. Currently, the corrected visual acuity is 1.2/1.2, and the IOP is 16/19 mmHg.

Patient IV-1, the proband, was referred to our hospital at 44 years of age with unexplained ocular hypertension and an abnormally narrow angle in the left eye. After ruling out diseases that cause narrow corner angles, such as Vogt-Koyanagi-Harada disease, the patient underwent cataract surgery for anterior lens deviation. IOP and anterior chamber depth quickly normalized after surgery, and visual acuity improved. In the following month, ocular hypertension developed in the right eye, prompting cataract surgery. Similar to the left eye, the right eye had an uneventful postoperative course. Lens cloudiness in both eyes was appropriate for age (Emery-Little grade 0-1). Currently, the patient has IOL donesis in both eyes. YAG laser capsulotomy was performed after cataract surgery because of the progression of posterior capsule opacification and anterior capsule contraction. Currently, the patient’s corrected visual acuity is 1.2/1.2 and IOP is 14/14 mmHg.

The four patients had no flat corneas or irises, abnormal axial lengths, evidence of retinal degeneration, or history of retinal detachment (Supplementary Table 1). A detailed physical examination by a cardiologist revealed that none of the family members had aortic root enlargement, aortic dissection, mitral valve deviation, thrombosis, patent ductus arteriosus, left superior vena cava remnant, or tricuspid or mitral valve regurgitation, as observed in MFS and WMS. Patient III-2 had mild mitral regurgitation, which does not require treatment.

A detailed physical examination was performed by an orthopedic surgeon. None of the patients had wrist or thumb signs, pectus carinatum, pectus excavatum, chest asymmetry, hindfoot deformity or flat feet, acetabular protrusion, reduced upper-to-lower segment ratio of the arm, increased arm span/height ratio, facial features, short stature, brachycephaly, joint stiffness, or arachnodactyly. Patients II-2 (158 cm) and III-3 (149 cm) were relatively short but not significantly shorter than the Japanese average for the same age group. Patient III-3 had a history of total hip arthroplasty. Patients II-2 and III-2 had mild, but not pathological scoliosis. Only patient II-2 showed a reduced elbow extension. The Bouchard nodes showed suspected deformities for patients II-2 and III-2. The clinical characteristics of the affected family members are summarized in Supplementary Table 1.

Other family members were interviewed regarding their medical histories. This questioning revealed that the proband’s great-grandfather (I-1) lost sight due to glaucoma in his 50 s and died due to old age. His medical history led to the conclusion that EL caused glaucoma. The maternal grandfather of the proband (II-1), who was strongly suspected to be a carrier of the variant responsible for EL, had no ocular symptoms and died when he was 47 years of age from multi-organ failure due to dental infection. Patient IV-1 has three sons in their 20 s. Patient III-3 has a son who is in his 30 s. No ophthalmological problems were noted in these younger family members, and genetic testing has not yet been conducted. No family history of abnormal stature or sudden death exists.

### Genetic analysis

**Fig. 3:**
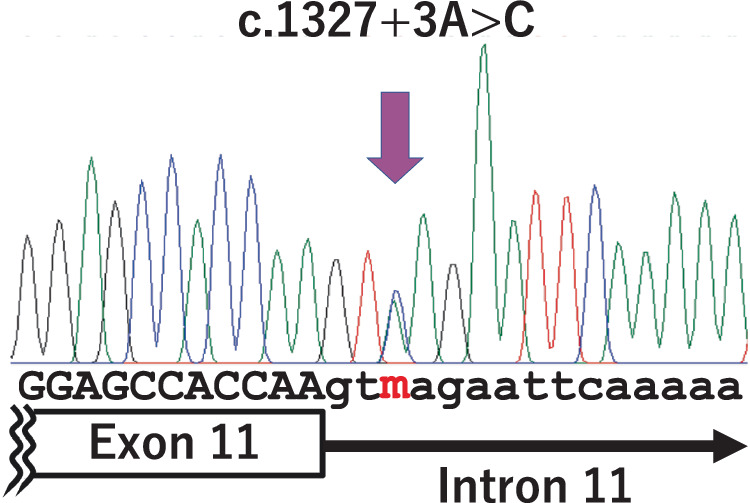
Novel intronic variant of *FBN1* identified in this study. Electropherograms from PCR direct sequencing of the *FBN1* exon 11–intron 11 boundary for patient III-3 are shown

WES did not identify coding variants of genes previously associated with hereditary lens luxation that were shared by IV-1 and III-2. In contrast, IV-1 and III-2 were both heterozygous for an A-to-C substitution in the third nucleotide of the 11th intron of *FBN1* (NM_000138.5). No variants at the nucleic acid position have been reported in the literature or registered in databases. The variant c.1327+3A>C was predicted to have high deleteriousness, with a CADD score of 26.1, and to influence the function of the splicing donor, with a delta score of 0.93 for donor loss in SpliceAI and a donor score of −5.484 in MMSplice. Sanger sequencing confirmed that the other affected individuals in the family (II-2 and III-3) were heterozygous for the same variant (Fig. 3).

### Analyses of *FBN1* transcripts from PBMCs

**Fig. 4:**
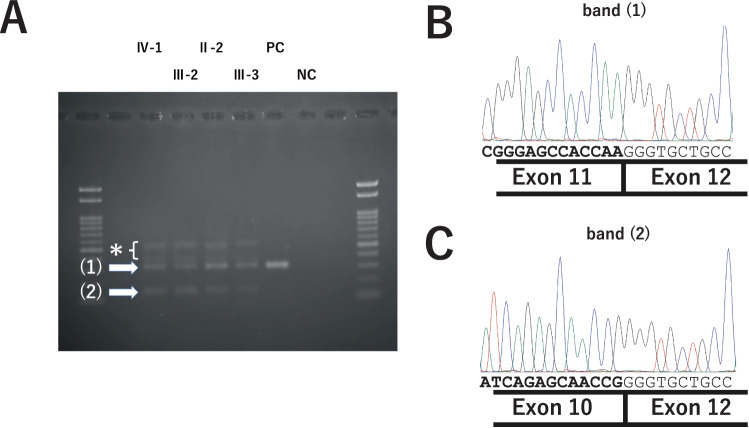
Analysis of the splicing of *FBN1* pre-RNAs from the patients’ peripheral blood mononuclear cells. **A** Image of the RT-PCR products resolved by gel electrophoresis. PC positive control (healthy adult), NC negative control (distilled water). In addition to the amplicon of the same size as that seen in PC (1), aberrant bands, including smaller (2) and larger smeary bands (*) were observed in patients IV-1, III-2, II-2, and III-3. Sanger sequencing result of amplicon (1) (**B**) and amplicon (2) (**C**)

A single-sized amplicon was detected using RT-PCR of the *FBN1* transcript in PBMCs from a healthy individual. Sanger sequencing of the amplicon verified the specific amplification of the target gene and the correctness of splicing. Multiple bands that migrated differently in the gel were observed for the four affected individuals (Fig. 4A). Sanger sequencing revealed that the smaller band corresponded to *FBN1* transcripts from which exon 11 was entirely deleted (Fig. 4B, C), and the smeared bands (* in Fig. 4A) were derived from complexes of amplicon strands with or without exon 11 skipping.

### Splicing analysis using the minigene model

**Fig. 5:**
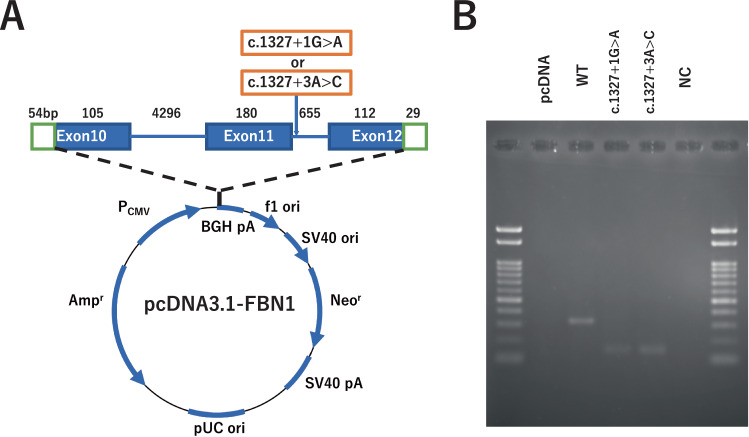
Analysis of splicing patterns using the *FBN1* minigene model. **A** Structure of pcDNA3.1-FBN1 harboring partial exon 10, intron 10, exon 11, intron 11, and partial exon 12 of the *FBN1* gene. **B** Results of RT-PCR for RNAs expressed from wild-type (WT) or mutant (MT) minigene constructs in HEK293 cells. NC negative control (distilled water)

To verify whether the aberrant splicing of *FBN1* observed in the patient transcripts was caused solely by c.1327+3A>C, we performed in vitro minigene experiments (Fig. 5A). We used three minigene constructs corresponding to WT, c.1327+3A>C, and c.1327+1G>A, a variant previously found in independent patients with EL. Agarose gel electrophoresis of the RT-PCR products revealed that the transcripts from the WT minigene exhibited normal splicing, whereas transcripts from both the c.1327+3A>C and c.1327+1G>A models lacked exon 11, as observed in the patient PBMC transcripts (Fig. 5B).

## Discussion

In 2010, the Ghent nosology for MFS was updated to classify patients with both EL and *FBN1* gene mutations as having MFS if the gene mutation was previously associated with aortic dilation or dissection [7]. According to the revision, many patients previously diagnosed with IEL are now diagnosed with MFS. It has been pointed out that only 34% of IEL patients having *FBN1* mutations but lacking the systemic features of MFS would remain as IEL sufferers after reclassification under the new criteria and that approximately 60% of *FBN1* mutations that had been considered “EL mutations” would not qualify as such [23].

IEL can be resolved by surgery and is not life-threatening. Therefore, especially in sporadic or mild cases of IEL, the motivation for genetic testing may be lower than that in patients with MFS. Many patients with IEL caused by *FBN1* mutations possibly remain undiagnosed, which has delayed the accumulation of information regarding EL mutations. The c.1327+3A>C variant is a novel variant not registered in gnomAD or ClinVar, and its association with aortic dilation/dissection has not yet been reported. In this study, we confirmed that all living family members with EL or a history of diseases that could be considered EL were adults and had no other systemic symptoms associated with MFS. Therefore, the family can be reasonably affected by IEL. The maternal grandfather of proband (II-1), who died at an age close to the proband’s onset of EL, had no eye abnormalities. Except for II-1, all family members who shared the c.1327+3A>C mutation, including I-1, the presumed founder of this mutation, had a history of EL or EL-related complications. However, we could not conclude that II-1 was non-penetrant, as some affected family members developed EL after the age of 60 years.

Two previous reports describe patients with EL having the same variant at the 5′ end of the *FBN1* intron 11 [21, 22]. The variant identified in the earlier patients was a single-nucleotide substitution within the splice donor consensus sequence (c.1327+1G>A). The patients with EL included a European female diagnosed at 41 years of age and a 68-year-old male Chinese and his 47-year-old daughter. The European female was described as having IEL [21] based on the revised Ghent nosology. The Chinese parent–offspring pair-sharing phenotypes of brachydactyly, EL, and short stature were described as patients with Weill–Marchesani-like syndrome [22]. In this Chinese family, the variant was further transmitted to a 17-year-old boy who was phenotypically normal. Thus, although incomplete penetrance and variable expressivity must be considered, all c.1327+1G>A carriers in previous reports lacked cardiovascular or skeletal abnormalities, which are diagnostic features of MFS.

The results of our minigene experiments suggest that both c.1327+1G>A and c.1327+3A>C lead to the skipping of exon 11. c.1327+3A>C, which co-segregated with EL in the studied family, is a substitution of the third nucleotide of an intron from purine to pyrimidine. The third nucleotide of U2-type introns of human genes is a purine nucleotide at frequencies >90%, and there are conserved nucleotide combinations at the +3 and +5 positions (+3A to +5G and +3G to +5A) [24]. Intron 11 of *FBN1* has the +3A to +5G combination at the 5′ end (Fig. 3). SpliceAI and MMsplice scores indicated that c.1327+3A>C affects the splicing of the 11th intron of *FBN1*, similar to c.1327+1G>A, which has previously been linked to EL. The sequencing results of the aberrant RT-PCR bands were consistent with this hypothesis. Collectively, these results suggest that the c.1327+3A>C mutation in *FBN1* is a newly identified mutation that causes EL.

Exon 11 of *FBN1* is 180 bases long. Skipping this exon may result in an in-frame deletion of 60 amino acids. The skipping of exon 11 is expected to alter amino acid translation at the junctions between exon 10–11 (GAG; glutamate) and 11–12 (AGG; arginine) and to reorganize them into a new codon at the exon 10–12 junction (GGG) encoding glycine (Supplementary Fig. 1). A large portion of the 60 amino acids encoded in exon 11, which was missing in the mutant *FBN1* protein of the studied patient, corresponded to the proline-rich region (Supplementary Fig. 1) [25]. Sengle et al. reported a family with WMS2 caused by a deletion of a genomic region involving FBN1 exons 10–12, resulting in an in-frame deletion [26]. In this previous study, the protein expression of *Fbn1* transcripts lacking exon 10–12 was confirmed in knock-in mouse experiments. In addition, biochemical assays revealed that the deleted N-terminal portion of the Fbn1 protein harboring transforming growth factor-β-binding-like (TB), proline-rich, and epidermal growth factor-like domains encoded in the three exons (Supplementary Fig. 1) functions as a binding site for A Disintegrin and Metalloproteinase with Thrombospondin motifs (ADAMTS)-like (Adamtsl) proteins. Fbn1, Adamtsl, and Adamts-10 form ternary complexes. *ADAMTS-10* is responsible for the autosomal recessive form of WMS (WMS1; OMIM #277600). Among the shared WMS manifestations of the affected family members in the previous study, only EL was observed in the family in the current study. The mutant FBN1, in which only amino acids coded in exon 11 are deleted, might maintain at least a partial interaction with other proteins and does not cause skeletal and skin symptoms characteristic of WMS. In contrast, a 12-base deletion near the 5’ end of *FBN1* exon 11 (rs672601352), which is expected to result in an in-frame deletion of 4 amino acids within the TB domain, has been identified in MFS and classified as a pathogenic/likely pathogenic variant (Supplementary Table 3). Hence, the phenotypes resulting from in-frame deletions as well as in-frame exon skipping variants of *FBN1*, even within this narrow region, may differ considerably depending on the size and area of the protein deletion.

The proline-rich region of the *FBN1* is, like those seen four times in the dystrophin protein, thought to act as a “hinge” in the folding of the protein and in organizing fibrillin-rich microfibrils [27]. The distal rod domain, including the third hinge region (hinge 3) of dystrophin, is a hotspot for in-frame deletions that cause Becker muscular dystrophy (BMD). It has been reported that the absence or presence of hinge 3 in the shortened protein, rather than the size of the in-frame deletion, significantly impacts the severity of BMD [28]. Given that the effect of hinge 3 deletion on the shortened dystrophin protein is thought to be due to a change in its primary structure, it is assumed that the FBN1 monomer lacking the proline-rich region will be similarly affected, which in turn will interfere with its function as a multimer. Since no previous studies have precisely elucidated the function of the proline-rich region in FBN1, further research is required to understand the impact of in-frame deletions on protein function.

Both haploinsufficiency (HI) and dominant-negative (DN) effects have been recognized as the pathogenic mechanisms of *FBN1* mutations in MFS [29]. FBN1 is a structural protein that functions as a multimer and, like several collagens, has the characteristics of a protein whose mutations cause an AD genetic disease through the DN effect. Missense mutations, in-frame insertions or deletions, and in-frame exon skipping variants are known to cause MFS with DN effects [30]. Although not molecularly validated for individual mutations, it is generally recognized that *FBN1* variants that create a premature termination codon (PTC), including nonsense variants, out-of-frame insertions or deletions, variants within consensus sequences in splice donor/acceptor sites, or in the initiation codon resulting in out-of-frame exon skipping, exhibit pathogenicity for MFS based on HI. Several studies have revealed that patients with *FBN1* mutations that cause PTC have a lower risk of EL than those with DN mutations, represented by missense mutations involving cysteine residues [31–33]. Since the revision of Ghent nosology, information on FBN1 variants for IEL has been accumulating, and many of these variants are missense variants that substitute or create cysteine residues and are not expected to exert the HI effect [21, 34–41]. Most pathogenic or likely pathogenic variants of *FBN1* exon 11 currently registered in ClinVar are nonsense or frameshift variants found in patients with MFS or family/isolated thoracic aortic aneurysms (Supplementary Table 3). Taken together, we hypothesize that the underlying mechanism of the EL phenotype in the studied family is more likely due to the DN effect of the mutant protein rather than HI.

Because of our experimental design and the paucity of patient specimens other than PBMCs, we cannot exclude the possibility of HI caused by reduced stability or translation efficiency of the mutant transcripts, specifically in the zonule tissue. We also acknowledge the possibility that c.1327+3A>C and c.1327+1G>A induce abnormal splicing with retention of intron 11, involving additional exons or using cryptic splice sites. A few of the resulting transcripts may have a PTC because of the translation of the retained intron or a frameshift. The proportions of in-frame and frameshift transcripts from these two mutant alleles may differ, especially among the organs affected by IEL, MFS, and WMS. Therefore, further information on variants around the exon 11–intron 11 boundary is required to elucidate the genotype–phenotype correlation.

## Conclusion

Here, we report familial patients with IEL caused by a novel, intronic, single-nucleotide variant within the *FBN1* gene. Further elucidation of the impact of deletion of the proline-rich region on FBN1 function is expected to provide insights into the pathogenesis of EL in this family as well as genotype–phenotype correlations.

## Supplementary information

Supplementary Table 1

Supplementary Table 2

Supplementary Table 3

Supplementary Figure 1

## Data Availability

Data are available upon reasonable request to the corresponding author.
