# Supplementary material for: Novel FBN1 intron variant causes isolated ectopia lentis via in-frame exon skipping
Source: J Hum Genet. 2025 Feb 13;70(4):199–205. doi: 10.1038/s10038-025-01318-0 (PMC11882438; doi:10.1038/s10038-025-01318-0)
Supplement: Supplementary file 1 — Supplementary Table 1 [file 10038_2025_1318_MOESM1_ESM.pdf]

Supplementary Table 1. Clinical manifestations of four family members affected with ectopia lentis.

	Family members	**II-2**	**III-2**	**III-3**	**IV-1**
**General**					
	Sex	Male	Female	Female	Female
	Genotype (*FBN1*)	WT/c.1327+3A>C	WT/c.1327+3A>C	WT/c.1327+3A>C	WT/c.1327+3A>C
	Age (years)	90	71	63	44
**Eyes**					
	Age at diagnosis of ectopia lentis (R/L, years)	57/64	55/56	62/55	44/44
	BCVA (R/L)	0.7/0.1	1.2/1.2	1.2/1.2	1.2/1.2
	Ectopia lentis (R/L)	+/±	+/+	+/+	+/+
	IOP (R/L, mmHg)	13/14	20/19	16/19	14/14
	Glaucoma	-	+	-	-
	Axial length (R/L, mm)	22.57/22.83	23.72/24.74	23.17/22.95	23.81/23.88
	Retinal detachment	-/-	-/-	-/-	-/-
	Corneal thickness (R/L, μm)	601/858	600/609	570/566	581/584
	Corneal power (R/L, D)	44.5/45.8	44.6/44.5	44.8/44.9	43.1/43.2
	Optic Disc Features		L) Cupping		
	Other Ophthalmic Features	L) Corneal endothelial failure	RNFL thinning		
**Cardiovascular system**					
	Aortic root dilatation/dissection	-	-	-	-
	Mitral valve prolapse	-	-	-	-
	Thrombosis	-	-	-	-
	Patent ductus arteriosus	-	-	-	-
	Persistent left superior vena cava	-	-	-	-
	Tricuspid/Mitral regurgitation	-	Mitral regurgitation(±)	-	-
	Other abnormal findings and ages at diagnosis (years)	-	-	-	-
**Skeletal system**					
	Thumb/wrist sign	-	-	-	-
	Funnel chest/Pectus carinatum	-	-	-	-
	Hallux valgus	-	-	-	-
	High arm-span to height ratio	-	-	-	-
	Height (cm)	154	161	148	161
	Arm span (cm)	158	161	149	162
	Arm span/height ratio	1.03	1	1.01	1.01
	Short stature	±	-	±	-
	Scoliosis/kyphosis	Scoliosis(±)	Scoliosis(±)	-	-
	Reduced elbow joint extension	+	-	-	-
	Characteristic facial features	-	-	-	-
	Brachycephaly	-	-	-	-
	Stiff joints	-	-	-	-
	Arachnodactyly	Bouchard's node(±)	Bouchard's node(±)	-	-
	Other abnormal findings and ages at diagnosis (years)	-	-	THA (62)	-

BCVA, best corrected visual acuity; F, female; HM, hand movement; IOP, intraocular pressure; L, left; LP, light perception; M, male; NA, not available; R, right RNFL, retinal nerve fiber layer thickness; THA,Total Hip Arthroplasty; WT, wildtype.
