# Supplementary material for: Novel FBN1 intron variant causes isolated ectopia lentis via in-frame exon skipping
Source: J Hum Genet. 2025 Feb 13;70(4):199–205. doi: 10.1038/s10038-025-01318-0 (PMC11882438; doi:10.1038/s10038-025-01318-0)
Supplement: Supplementary file 2 — Supplementary Table 2 [file 10038_2025_1318_MOESM2_ESM.pdf]

Supplementary Table 2. Sequences of the primers.

Primer pairs	Direction	Sequence (5' – 3')
Pr1	Forward	TAATTCCTGGGAGACCAGAATATCC
	Reverse	CTGGAGAATAGGAAGCCTCCC
Pr2	Forward	CTGTCGCCCCTGAGATGTGT
	Reverse	TGTTGCACTCACACCGGTAACTC
Pr3	Forward	TACCGAGCTCGGATCAGCTGCCACAGTCCATAACCAA
	Reverse	CTGGACTAGTGGATCAACCCTTTGTTGCACTCACACC
Pr4	Forward	CCACCAA**A**TAAGAATTCAAAAATCAT
	Reverse	ATTCTTA**T**TTGGTGGCTCCCGAGATG
Pr5	Forward	ACCAAGT**C**AGAATTCAAAAATCATCT
	Reverse	GAATTCT**G**ACTTGGTGGCTCCCGAGA

Note:

Pr1: Primers for PCR of genomic DNA around *FBN1* exon 11 – intron 11 boundary.

Pr2: Primers for amplification of a fragment encompassing 3' part of exon 10, exon 11 and 5' part of exon 12 of *FBN1* mRNA in RT-PCR.

Pr3: Primers for the construction of the *FBN1* minigene. 5' additional sequences homologous to the pcDNA3.1(+) vector are underlined.

Pr4, Pr5: Primers for the site-directed mutagenesis of c.1327+1 G>A (Pr4) and of c.1327+3 A>C (Pr5). Target nucleotide positions are in bold.
