# Supplementary material for: Novel FBN1 intron variant causes isolated ectopia lentis via in-frame exon skipping
Source: J Hum Genet. 2025 Feb 13;70(4):199–205. doi: 10.1038/s10038-025-01318-0 (PMC11882438; doi:10.1038/s10038-025-01318-0)
Supplement: Supplementary file 3 — Supplementary Table 3 [file 10038_2025_1318_MOESM3_ESM.pdf]

Supplementary Table 3. Pathegenic, Likely pathogenic and Pathogenic/Likely pathogenic variants within *FBN1* intron10, exon11 and intron11 found in ClinVar.

Location in *FBN1*	Nucleotide change	Protein change	Molecular consequence	dbSNP ID	ClinVar Accession	Germline classification	Condition(s)
intron10	c.1147+1G>A		splice donor variant	rs2141335918	VCV000982351	Likely pathogenic	Isolated thoracic aortic aneurysm
intron10	c.1147+2T>A		splice donor variant	rs2141335915	VCV001481304	Likely pathogenic	Familial thoracic aortic aneurysm and aortic dissection|Marfan syndrome
intron10	c.1148-2A>G		splice acceptor variant	rs397515756	VCV000042283	Pathogenic/Likely pathogenic	Marfan Syndrome/Loeys-Dietz Syndrome/Familial Thoracic Aortic Aneurysms and Dissections|Marfan syndrome|Familial thoracic aortic aneurysm and aortic dissection|not provided|Marfan syndrome
intron10	c.1148-2A>C		splice acceptor variant	rs397515756	VCV000199965	Pathogenic	not provided
intron10	c.1148-1G>A		splice acceptor variant	rs1555400431	VCV000450336	Likely pathogenic	not provided
intron10	c.1148-1G>C		splice acceptor variant		VCV001732987	Likely pathogenic	Familial thoracic aortic aneurysm and aortic dissection
exon11	c.1155_1165del	p.Phe385fs	frameshift variant		VCV002025064	Pathogenic	Marfan syndrome|Familial thoracic aortic aneurysm and aortic dissection
exon11	c.1156_1167del	p.Asn386_Cys389del	inframe_deletion	rs672601352	VCV000162038	Pathogenic/Likely pathogenic	Marfan syndrome
exon11	c.1160del	p.Lys387fs	frameshift variant		VCV003074950	Likely pathogenic	Marfan syndrome
exon11	c.1167C>A	p.Cys389Ter	nonsense	rs746201757	VCV000549006	Likely pathogenic	Marfan syndrome
exon11	c.1169_1170del	p.Ser390fs	frameshift variant	rs2141331190	VCV001329353	Likely pathogenic	Familial thoracic aortic aneurysm and aortic dissection
exon11	c.1187del	p.Pro396fs	frameshift variant		VCV003230406	Pathogenic	Familial thoracic aortic aneurysm and aortic dissection
exon11	c.1188_1206del	p.Gly397fs	frameshift variant		VCV002945420	Pathogenic	Marfan syndrome|Familial thoracic aortic aneurysm and aortic dissection
exon11	c.1192A>T	p.Arg398Ter	nonsense	rs397515754	VCV000042281	Pathogenic	Marfan syndrome
exon11	c.1200del	p.Glu400fs	frameshift variant		VCV002114632	Pathogenic	Marfan syndrome|Familial thoracic aortic aneurysm and aortic dissection
exon11	c.1211del	p.Pro404fs	frameshift variant	rs112289537	VCV000036033	Pathogenic/Likely pathogenic	not provided|Marfan syndrome
exon11	c.1216del	p.Gly407fs	frameshift variant	rs2141331057	VCV001422238	Pathogenic	Familial thoracic aortic aneurysm and aortic dissection|Marfan syndrome|Familial thoracic aortic aneurysm and aortic dissection
exon11	c.1220del	p.Gly407fs	frameshift variant		VCV002943895	Pathogenic	Marfan syndrome|Familial thoracic aortic aneurysm and aortic dissection
exon11	c.1238_1239insAG	p.Pro414fs	frameshift variant	rs2043800325	VCV000985105	Pathogenic	Inborn genetic diseases
exon11	c.1245del	p.Pro416fs	frameshift variant	rs2141331000	VCV001075494	Pathogenic	Marfan syndrome|Familial thoracic aortic aneurysm and aortic dissection
exon11	c.1270C>T	p.Gln424Ter	nonsense	rs1439533354	VCV000549009	Pathogenic	Marfan syndrome
exon11	c.1285C>T*	p.Arg429Ter	nonsense	rs112645512	VCV000180351	Pathogenic/Likely pathogenic	Marfan syndrome|Marfan syndrome|Familial thoracic aortic aneurysm and aortic dissection|not provided|Marfan Syndrome/Loeys-Dietz Syndrome/Familial Thoracic Aortic Aneurysms and Dissections|MASS syndrome|Stiff skin syndrome|Acromicric dysplasia|Ectopia lentis 1, isolated, autosomal dominant|Marfan syndrome|Weill-Marchesani syndrome 2, dominant|Geleophysic dysplasia 2|Progeroid and marfanoid aspect-lipodystrophy syndrome|Familial thoracic aortic aneurysm and aortic dissection
exon11	c.1297G>T	p.Glu433Ter	nonsense	rs2141330884	VCV001098807	Likely pathogenic	Marfan Syndrome/Loeys-Dietz Syndrome/Familial Thoracic Aortic Aneurysms and Dissections
exon11	c.1302T>A	p.Tyr434Ter	nonsense	rs1566916969	VCV000571414	Pathogenic	Familial thoracic aortic aneurysm and aortic dissection|Marfan syndrome
exon11	c.1318G>T	p.Glu440Ter	nonsense	rs1555400406	VCV000549010	Pathogenic	Marfan syndrome
intron11	c.1328-1G>A		splice acceptor variant		VCV001770046	Pathogenic	Familial thoracic aortic aneurysm and aortic dissection

* Although identified in a faimity with isolated ectopia lentis, this vatiant is not difined as IEL mutation based on the revised Ghent nosology.
