# Supplementary material for: Novel FBN1 intron variant causes isolated ectopia lentis via in-frame exon skipping
Source: J Hum Genet. 2025 Feb 13;70(4):199–205. doi: 10.1038/s10038-025-01318-0 (PMC11882438; doi:10.1038/s10038-025-01318-0)
Supplement: Supplementary file 4 — Supplementary Figure 1 [file 10038_2025_1318_MOESM4_ESM.pdf]

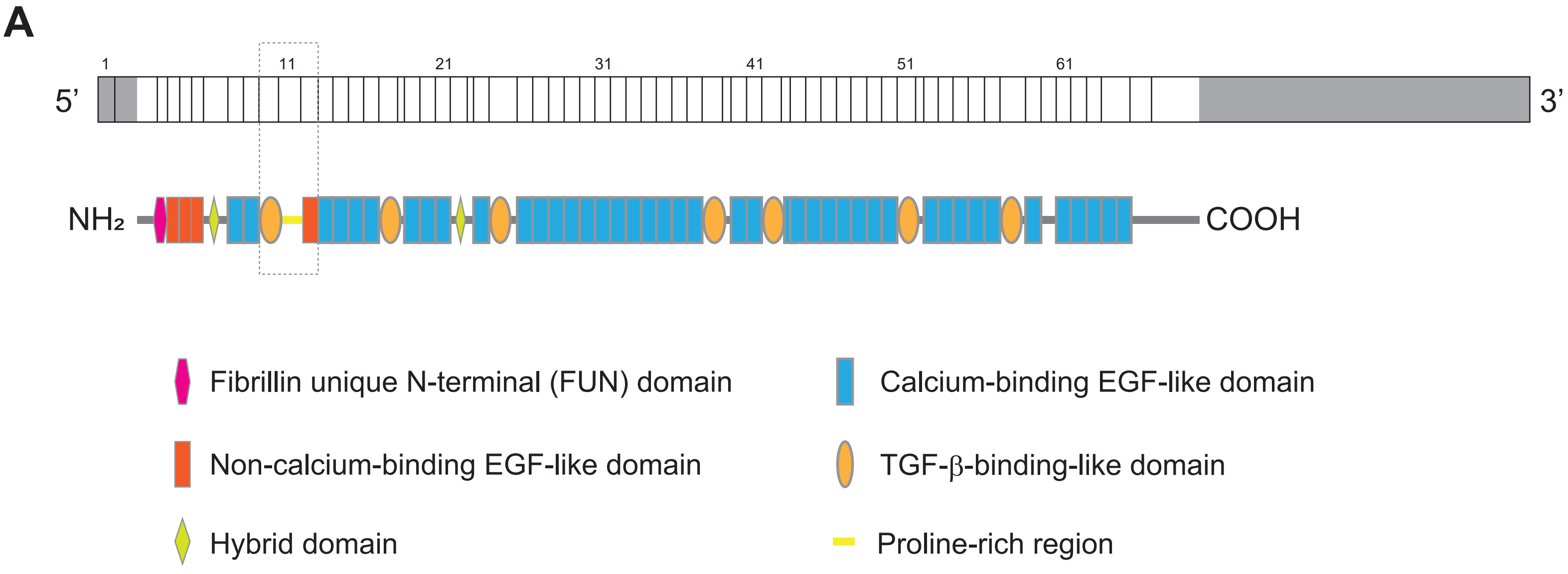

B

```
        Exon9(partly)                           Exon10
961     ACC TCT CCA GAT GGT ACC AGA TGC ATA GAT GTT CGC CCA GGA TAC TGT TAC ACA GCT CTG
321     Thr Ser Pro Asp Gly Thr Arg Cys Ile Asp Val Arg Pro Gly Tyr Cys Tyr Thr Ala Leu

1021    ACA AAC GGG CGC TGC TCT AAC CAG CTG CCA CAG TCC ATA ACC AAA ATG CAG TGC TGC TGT
341     Thr Asn Gly Arg Cys Ser Asn Gln Leu Pro Gln Ser Ile Thr Lys Met Gln Cys Cys Cys

1081    GAT GCC GGC CGA TGC TGG TCT CCA GGG GTC ACT GTC GCC CCT GAG ATG TGT CCC ATC AGA
361     Asp Ala Gly Arg Cys Trp Ser Pro Gly Val Thr Val Ala Pro Glu Met Cys Pro Ile Arg
                 Exon11
1141    GCA ACC GAG GAT TTC AAC AAG CTG TGC TCT GTT CCT ATG GTA ATT CCT GGG AGA CCA GAA
381     Ala Thr Glu Asp Phe Asn Lys Leu Cys Ser Val Pro Met Val Ile Pro Gly Arg Pro Glu

1201    TAT CCT CCC CCA CCC CTT GGC CCC ATT CCT CCA GTT CTC CCT GTT CCT CCT GGC TTT CCT
401     Tyr Pro Pro Pro Pro Leu Gly Pro Ile Pro Pro Val Leu Pro Val Pro Pro Gly Phe Pro

1261    CCT GGA CCT CAA ATT CCG GTC CCT CGA CCA CCA GTG GAA TAT CTG TAT CCA TCT CGG GAG
421     Pro Gly Pro Gln Ile Pro Val Pro Arg Pro Pro Val Glu Tyr Leu Tyr Pro Ser Arg Glu
                 Exon12
1321    CCA CCA AGG GTG CTG CCA GTA AAC GTT ACT GAT TAC TGC CAG TTG GTC CGC TAT CTC TGT
441     Pro Pro Arg Val Leu Pro Val Asn Val Thr Asp Tyr Cys Gln Leu Val Arg Tyr Leu Cys

1381    CAA AAT GGA CGC TGC ATT CCA ACT CCT GGG AGT TAC CGG TGT GAG TGC AAC AAA GGG TTC
461     Gln Asn Gly Arg Cys Ile Pro Thr Pro Gly Ser Tyr Arg Cys Glu Cys Asn Lys Gly Phe
                                                          Exon13(partly)
1441    CAG CTG GAC CTC CGT GGG GAG TGT ATT GAT GTT GAT GAA TGT GAG AAA AAC CCC TGT GCT
481     Gln Leu Asp Leu Arg Gly Glu Cys Ile Asp Val Asp Glu Cys Glu Lys Asn Pro Cys Ala
```

Supplementary Figure 1. Domain organization of FBN1 and corresponding exons.
(A) Upper; 66 exons in *FBN1* mRNA (NM_000138.5). 5’ and 3’ untranslated regions are shown with gray boxes. Lower; domains of FBN1 protein. Information on the domains, except for the proline-rich region, were obtained from Uniprot (https://www.uniprot.org/uniprotkb/P35555/entry). Nucleotide and amino acid sequences for the area enclosed by a dotted rectangle are detailed in B.
(B) Nucleotides and amino acid sequences around *FBN1* exon11. Nucleic acids of exon 11 were shown in bold. Peptides of the TGF-β-binding protein-like domain coded in exons 10 and 11 and the non-calcium binding EGF-like domain coded in exons 12 and 13 are highlighted in orange, and reddish-brown respectively. Proline residues within the proline-rich region are highlighted in yellow. Nucleotides involved in the codon spanning the exon10-12 junction and those deleted in a known in-frame deletion variant (rs672601352) are indicated by red and blue letters, respectively.
